# Comparing data-driven physiological denoising approaches for resting-state fMRI: implications for the study of aging

**DOI:** 10.3389/fnins.2024.1223230

**Published:** 2024-02-06

**Authors:** Ali M. Golestani, J. Jean Chen

**Affiliations:** ^1^Department of Physics and Astronomy, University of Calgary, Calgary, AB, Canada; ^2^Department of Oncology, Cumming School of Medicine, University of Calgary, Calgary, AB, Canada; ^3^Rotman Research Institute at Baycrest, Toronto, ON, Canada; ^4^Department of Medical Biophysics, University of Toronto, Toronto, ON, Canada; ^5^Department of Biomedical Engineering, University of Toronto, Toronto, ON, Canada

**Keywords:** fMRI, resting-state, physiological noise, data-driven, brain connectivity

## Abstract

**Introduction:**

Physiological nuisance contributions by cardiac and respiratory signals have a significant impact on resting-state fMRI data quality. As these physiological signals are often not recorded, data-driven denoising methods are commonly used to estimate and remove physiological noise from fMRI data. To investigate the efficacy of these denoising methods, one of the first steps is to accurately capture the cardiac and respiratory signals, which requires acquiring fMRI data with high temporal resolution.

**Methods:**

In this study, we used such high-temporal resolution fMRI data to evaluate the effectiveness of several data-driven denoising methods, including global-signal regression (GSR), white matter and cerebrospinal fluid regression (WM-CSF), anatomical (aCompCor) and temporal CompCor (tCompCor), ICA-AROMA. Our analysis focused on the consequence of changes in low-frequency, cardiac and respiratory signal power, as well as age-related differences in terms of functional connectivity (fcMRI).

**Results:**

Our results confirm that the ICA-AROMA and GSR removed the most physiological noise but also more low-frequency signals. These methods are also associated with substantially lower age-related fcMRI differences. On the other hand, aCompCor and tCompCor appear to be better at removing high-frequency physiological signals but not low-frequency signal power. These methods are also associated with relatively higher age-related fcMRI differences, whether driven by neuronal signal or residual artifact. These results were reproduced in data downsampled to represent conventional fMRI sampling frequency. Lastly, methods differ in performance depending on the age group.

**Discussion:**

While this study cautions direct comparisons of fcMRI results based on different denoising methods in the study of aging, it also enhances the understanding of different denoising methods in broader fcMRI applications.

## Introduction

It has become established that for a method aiming to quantify brain function, resting-state blood-oxygenation level-dependent (BOLD) fMRI (rs-fMRI) metrics is exquisitely sensitive to underlying brain physiology, embodied in such variables as cerebrovascular reactivity (Golestani et al., 2016; Chu et al., 2018; Tsvetanov et al., 2020). Moreover, physiological contributions from cardiac and respiratory frequencies constitute a major source of noise in the BOLD fMRI data (Liu, 2016). Providing that the sampling rate of the fMRI data is sufficiently high to faithfully capture the fundamental cardiac and respiratory frequencies, the time-locked components of these noise sources can be largely reduced by a notch filter (Chen et al., 2019). However, the sampling rate of a typical fMRI data is not sufficiently high, resulting in aliasing of the physiological signals into lower frequencies, rendering the application of notch filters impractical. Alternatively, methods have been developed to model and remove phase-locked physiological on the BOLD fMRI signal based on recorded physiological signals (Glover et al., 2000). For these purposes, cardiac and respiratory recordings should be obtained during the fMRI data acquisition. However, this can be challenging, unreliable, and in some cases impossible due to experimental limitations (Agrawal et al., 2020). Data-driven methods represent an alternative, whereby estimates of the physiological effects are generated from the fMRI data itself.

One of the oldest data-driven denoising approaches is to regress out the global BOLD signal (GS), calculated by averaging signals of the voxels within the brain. Global-signal regression (GSR) assumes that because the effect of physiological signals on the fMRI data is widespread, regressing out the GS will remove physiological and other noise sources. However, GSR remains controversial as it can also remove information relevant to brain function and connectivity (Liu et al., 2017). Brain states such as arousal and vigilance can also alter the GS through whole-brain effects (Wong et al., 2013; Gu et al., 2019). Nonetheless, GSR remains widely used for datasets with a high level of global noise (Chen et al., 2012), as it improves anatomical specificity of the connectivity maps (Fox et al., 2009) and increases the behavioral correlations with connectivity patterns (Li et al., 2019).

An alternative to GSR is to regress out the average signals derived only from the white matter (WM) and cerebrospinal fluid (CSF), where neuronal contributions are thought to be negligible (Bartoň et al., 2019). The WM-CSF regression approach found considerable application (Satterthwaite et al., 2013; Parkes et al., 2018; He et al., 2020; Scheel et al., n.d.). However, there is evidence that WM also contains information about brain function (Ding et al., 2013; Mazerolle et al., 2013; Peer et al., 2017; Wang et al., 2022). Moreover, the average signal across WM and CSF cannot account for regional-specific temporal variations of the physiological effects (Attarpour et al., 2021).

Alternatively, the CompCor family of methods applies principal component analysis (PCA) on a collection of signals from “noise” regions of interest (ROIs) to decompose them into uncorrelated components, such that only a specific number of components with the highest variance are removed (Behzadi et al., 2007). CompCor has two variants; anatomical CompCor (aCompCor) defines noise sources anatomically, by focusing on signals within WM and CSF anatomical masks, whereas temporal CompCor (tCompCor) defines noise sources temporally, by focusing on signals with high temporal standard deviation irrespective of their spatial origin. The aCompCor method is built into software packages as the CONN Toolbox (Whitfield-Gabrieli and Nieto-Castanon, 2012), and has been widely applied.

As a departure from these conventional model-driven methods, independent component analysis (ICA) has been used to spawn a family of techniques for extracting representatives of the physiological noise from the fMRI data (Thomas et al., 2002; Glasser et al., 2018; Golestani and Chen, 2022). ICA decomposes the fMRI data into spatially independent components, and assuming that the physiological noise and neuronally driven signals are spatially independent, ICA can separate them into different components. The noise-related components can be manually identified based on their spatial, temporal, and spectral features, especially as physiological noise can manifest as semi-regular head motion with distinct spatial patterns. Nonetheless, this process is subjective, which can introduce inter-cohort and inter-study variability. To address this issue, a series of ICA variants, such as ICA-FIX (semi-automatic noise classification) and ICA-AROMA (Automatic Removal of Motion Artifacts) have been developed that allow noise-related components to be spatially and temporally identified automatically (Salimi-Khorshidi et al., 2014; Pruim et al., 2015b). An advantage of ICA-AROMA over ICA-FIX is that the former makes use of spatiotemporal features to identify noise components, and thus does not require training of the noise classifiers with each new data set while retaining much of the functionally relevant correlational structure structure in the data. The performance of ICA-AROMA has been compared favorably against that of ICA-FIX (Pruim et al., 2015a; Dipasquale et al., 2017), and ICA-AROMA has become increasingly adopted in rs-fMRI analysis (Dipasquale et al., 2017; Cohen et al., 2021).

Despite widespread application of the data-driven noise removal methods, a thorough understanding of the efficacy of these noise removal techniques remains unclear. A number of recent studies made valuable contributions to such an understanding. For instance, Dipasquale et al. (2017) compared regression of motion parameters, the WM-CSF regression method, ICA-FIX, ICA-AROMA and multi-echo ICA, and found multi-echo ICA to be best at decoupling motion and neuronal effects. Scheel et al. compared censoring, GSR, ICA-AROMA (aggressive and non-aggressive) and SOCK (an AROMA variation), and found aggressive ICA-AROMA to provide the highest network reproducibility (Scheel et al., 2022). Moreover, Kassinopoulos et al. compared GSR and aCompCor in terms of a set of quality metrics including functional-connectivity repeatability and modularity (Kassinopoulos and Mitsis, 2022), and found a combination of aCompCor and GSR to provide the best outcomes. While these studies provide compelling comparisons of some of the methods introduced herein, they are based largely on repeatability of the connectivity maps instead of evaluations of the denoised signals themselves. In this sense, a systematic evaluation of efficacy faces a number of challenges. First, since the typical sampling rate of the fMRI data is above the Nyquist frequency of the respiration and heartbeat, these physiological signals alias into low frequencies, and therefore investigating the efficacy of the noise removal techniques is challenging. Thus, it is typically impossible to quantify the amount of major physiological noise sources in the signal. Second, the biggest roadblock for the application for rs-fMRI is in clinical translation, and it remains unclear whether superior sensitivity or repeatability translate into superior reflection of biological differences. Third, when assessing biological differences using rs-fMRI functional connectivity (fcMRI), there is no ground truth to compare against. In this regard, the sole dependence on conventional MRI quality metrics, such as sensitivity and reproducibility, may not be ideal for rs-fMRI, as the latter is expected to be variable with time (Tailby et al., 2015; Aedo-Jury et al., 2020). In this regard, it is intriguing to compare the effect of different denoising methods on a biologically interpretable effect such as that of age. Although age-related differences in fcMRI can be influenced by a combination of neuronal and physiological signals, comparing the effects of denoising methods on age-related differences can help to identify methodological sources of variability in research findings.

In this study, to address these challenges, we adopt the following methodological choices. First, we examined the BOLD signal’s frequency spectra. The signal power spectra have been used as quality metrics in previous work. For instance, using ultra-fast fMRI acquisitions, Agrawal et al. demonstrated the removal of physiological noise while retaining low-frequency signal power (Agrawal et al., 2020). On the other hand, using conventionally-sampled fMRI data, Dipasquale et al. (2017) showed that ICA-AROMA (non-aggressive) retained more low-frequency spectral power while more significantly reducing high-frequency power relative to other ICA-based methods, and in contrast to WM-CSF, which retained more higher-frequency noise. In our work, the power-spectral approach is facilitated by our high temporal-resolution fMRI data and simultaneously recorded physiological signals to accurately assess the location and contribution of physiological frequencies with minimal aliasing. Second, we examine the effect of age on fcMRI measures as a variable of the denoising method. It is important to note that signal and noise structures alter in aging (Van Dijk et al., 2012; Makedonov et al., 2013; Tsvetanov et al., 2015) including the frequency composition of the fMRI signal (Yang et al., 2018; Ao et al., 2022), which may be related to physiological processes and to neurovascular coupling changes (Yang et al., 2018). In fact, Geerligs et al. (2017) suggested that head-motion effects can change with age, and that motion regression may erase some of the age-relevant functional differences, highlighting the importance of appropriate physiological denoising. Third, instead of relying on sensitivity and reproducibility as metrics of denoising quality, we compared denoising methods by the extent to which the output of each denoising technique can alter age-related functional-connectivity (fcMRI) differences. We use these approaches to evaluate all data-driven denoising methods available through the commonly used fMRIPrep pipeline. We hypothesized that methods that removed more low-frequency signal power also resulted in a loss of sensitivity to age-related resting-state fcMRI (rs-fcMRI) differences.

## Method

### Participants and data acquisition

18 healthy young subjects (age = 26.7 ± 6.5 years, 9F/9M) and 18 healthy older subjects (age = 74.2 ± 7.0 years, 11F/7M) were imaged using a Siemens TIM Trio 3 T scanner (Siemens Healthineers, Erlangen, Germany). All participants were recruited from the Greater Toronto Area, and provided written informed consent as per the policy of our institutional research ethics board. rs-fMRI scans were collected with a 32-channel head coil using simultaneous multi-slice (SMS) echo-planar imaging (EPI) BOLD (TR/TE = 380/30 ms, FA = 40°, 21 5-mm slices, 64×64 matrix, 4x4x5 mm voxels, multiband factor = 3, 1,950 volumes, left–right phase encoding direction, in-plane acceleration of 2). During each scan, cardiac pulsation was recorded using the scanner pulse oximeter, whereas the respiratory signal was recorded using a Biopac^™^ system (Biopac Systems Inc. California, United States). A T1-weighted 3D anatomical data set (1 mm isotropic resolution) was also acquired for each participant (MPRAGE, TR = 2,400 ms, TE = 2.43 ms, field-of-view = 256 mm, voxel size = 1 mm isotropic, TI = 1,000 ms, BW = 180 Hz/vox).

### Data preprocessing and physiological denoising

**Figure 1:**
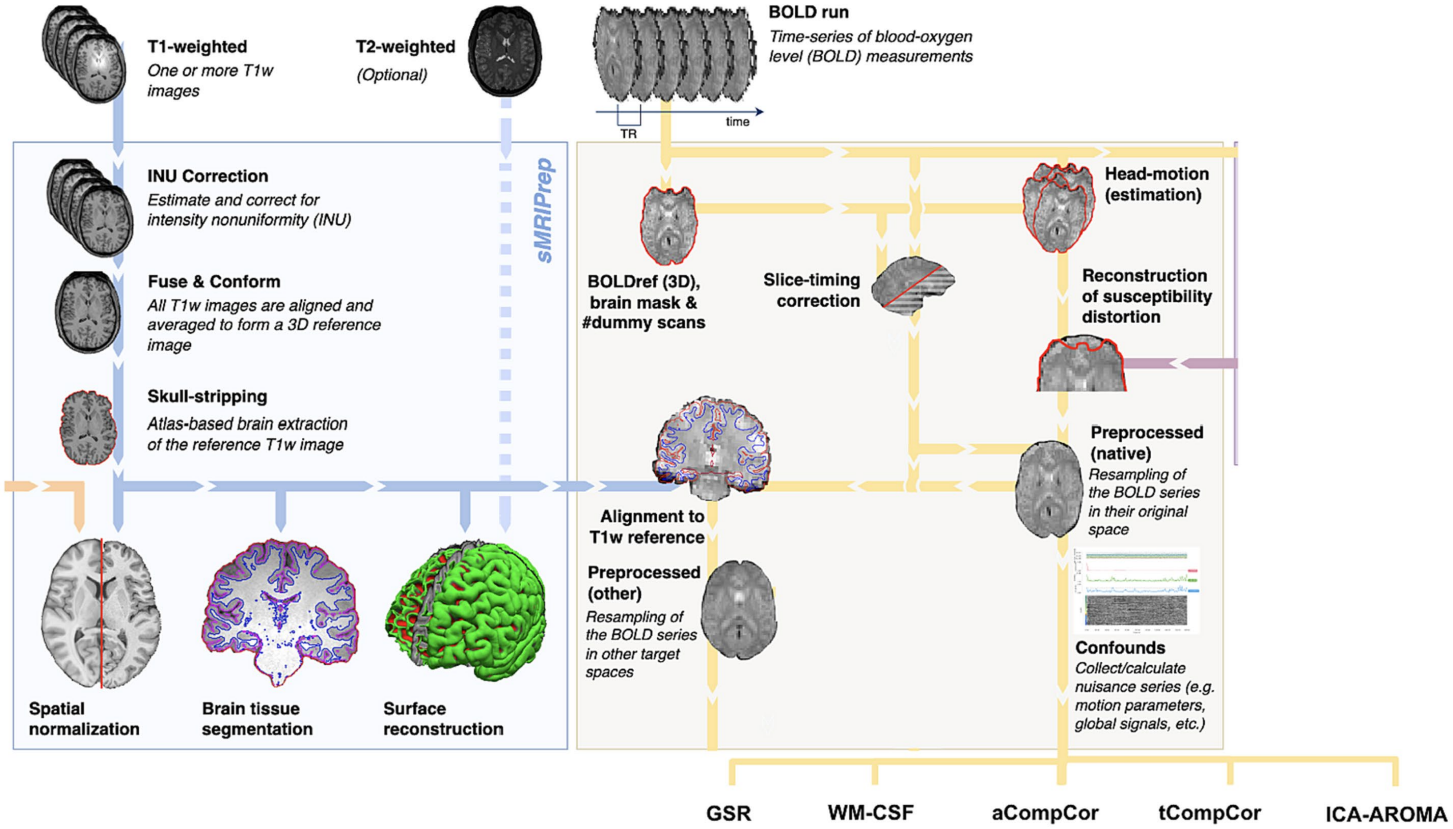
Summary of preprocessing steps (figure adapted from www.fmriprep.org). All illustrated preprocessing steps were applied to the fMRI and T1-weighted data as relevant. The tissue segmentation required for denoising is performed through FreeSurfer. In particular, the fMRI data underwent slice-timing correction, alignment to the T1 images, head motion estimation, susceptibility-distortion correction and confound estimation. The confounds produced by this pipeline are in turn used by some of the denoising strategies.

The anatomical segmentation was performed using fMRIPrep, as shown in Figure 1. Specifically, the T1 anatomical images were skull-stripped using a Nipype implementation of Advanced Normalization Tools (ANTs) (Avants et al., 2011), following which tissue segmentation was performed using FMRIB Software Library (FSL) and spatial normalization to standard MNI152 space was performed using ANTs. This step resulted in grey matter (GM), WM and CSF segmentations for each data set.

fMRI data preprocessing was performed using fMRIPrep (Version 22.0) (Esteban et al., 2019), and includes motion correction, spatial smoothing (5 mm FWHM), high-pass filtering (>0.01 Hz) and brain extraction. This is a common processing pipeline applied prior to all physiological denoising methods (no correction), though we also obtained framewise displacement (FD) time series for each data set based on Power et al. (2012). Temporal SNR (tSNR) on the preprocessed data (pre-denoising) was also computed for each subject, as the ratio between the mean and standard deviation of each time series. The preprocessed data was channeled separately through five data-driven denoising methods for comparison, as implemented through fMRIPrep. These methods include:

- Global signal regression (GSR): the global signal is generated by averaging signals within the brain mask (excluding CSF).
- White matter and CSF signal regression (WM-CSF): WM and CSF ROIs were obtained from the segmentation of the T1-weighted anatomical image of each subject, as explained above. WM and CSF signal is generated by averaging the signals within the anatomically-derived eroded masks.
- Anatomical CompCor (aCompCor): the five principal components (PCs) with the highest explained variance are used as confounders.
- Temporal CompCor (tCompCor): all PCs identified by tCompCor are regressed out.
- AROMA (ICA-AROMA): based on ICA, AROMA exploits a small set of four robust theoretically motivated temporal and spatial features associable to head motion, and all independent components exhibiting these features are identified as nuisance and removed. Though devised for head-motion correction, AROMA has found success for physiological denoising more broadly (Griffanti et al., 2017). In this work, the non-aggressive version of ICA-AROMA was implemented, and all identified components were regressed out.
- No correction: all methods were also compared to the case of no physiological denoising for reference.

Regressors of each method are estimated using fMRIprep (Esteban et al., 2019), publicly available at fmriprep.org. We did not regress out estimated motion parameters in addition to the noise regressors.

### Evaluation metrics

#### BOLD-signal spectral power

In order to identify the BOLD signal spectral peaks associated with time-locked physiological processes, subject-specific heartbeat and respiration frequencies were estimated based on the peak in the spectrum of their physiological recordings. Then, for each data set, the frequency spectra of fMRI signals are obtained, and then averaged separately across the gray matter and white matter, respectively, for each individual and the spectrum of the averaged signals is calculated. We computed the total fMRI signal power pre- and post-denoising in three frequency bands:

1. Cardiac band: a 0.1 Hz-wide band centered at the subject-specific heartbeat;
2. Respiratory band: a 0.2 Hz-wide band centered around each subject’s respiration frequency;
3. Low-frequency band: the frequency band between 0.01 and 0.1 Hz, commonly used for fcMRI assessments.

A successful denoising technique should maximally remove the frequencies associated with the cardiac and respiratory bands while preserving information in the low-frequency band. To evaluate the extent to which each denoising method alters the power contribution of these frequency bands, we computed the fractional power change with Eq. (1):


(1) $$\frac{P_{\mathrm{uncorrected}}-P_{\mathrm{corrected}}}{P_{\mathrm{uncorrected}}}$$

where *P*_uncorrected_ is the power of the fMRI signal before noise correction in one of the three frequency bands and *P*_corrected_ is the power of the fMRI signal after noise correction in these frequency bands. The use of fractional spectral-power change allows direct comparison across methods and age groups.

We subsequently performed formal statistical testing using multi-factor ANOVAs and paired/unpaired t-tests. ANOVAs were conducted using generalized linear mixed-effects models to analyze fractional spectral power, including age group, tissue type, and method as variables of interest. Each frequency band (low frequency, respiratory, and cardiac frequency bands) were analyzed separately. Follow-up *t*-tests were performed to compare fractional power changes associated with each factor. All comparisons were corrected for false discovery using the Benjamini-Hochberg method (Benjamini and Hochberg, 1995). All statistical analyses were performed using Matlab (Mathworks, Natick, United States).

#### rs-fcMRI metrics

For each processed fMRI dataset, template-based rotation (TBR) was used to generate fcMRI maps using Yeo functional network parcellations templates (Yeo et al., 2011). TBR is a new analytic technique that was designed for utilizing *a priori* functional parcellations to guide the analysis of individual sessions. TBR is similar to dual regression, but reverses the direction of prediction such that instead of individual volumes (time points) being predicted as linear sums of templates, templates are predicted as linear sums of volumes (Schultz et al., 2014). The TBR step produced fcMRI strengths quantified as t-maps, which were used in subsequent analyses without thresholding. First, we divided the GM into 200 ROIs based on the gradient weighted Markov Random field proposed by Schaefer et al. (2018), and generated Pearson’s correlation matrices for each subject and each denoising method. We then divided the ROIs based on the 7 functional networks, and computed the network-mean Pearson’s correlation within each network. A denoising method that exhibits a higher network-mean Pearson’s correlation tends to generate stronger resting-state functional connectivity. Secondly, we utilized functional-connectivity contrast (FCC) to evaluate network segregation. FCC is characterized as the z-statistic derived from the Wilcoxon rank-sum test, evaluating the null hypothesis that in a fcMRI map, connectivity values within a network and connectivity values between networks are drawn from distributions with identical medians (Kassinopoulos and Mitsis, 2022). Elevated FCC values indicate stronger connectivity within a network in contrast to between networks. We used FCC to compare the denoising methods in their effectiveness to distinguish resting-state networks from background processes. We evaluated FCC by individually computing it for each network and subsequently averaging the FCC values across the seven networks. Third, as a measure of the distinguishability of young and old groups based on their fcMRI maps, we also computed modularity, which assesses the strength of within-group coupling relative to the coupling between groups. To compute the modularity index, an adjacency matrix is generated by comparing the similarity of the connectivity maps using the cosine similarity index. Modularity (*Q*) is calculated using Eq. (2).


(2) $$Q=\frac{1}{4m}\underset{i,j}{\sum }\left(A_{ij}-\frac{k_{i}k_{j}}{2m}\right)s_{i}s_{j}$$

where *A* is the similarity matrix, m is the number of connections, *k*_i_ is the degree of node i (i.e., sum of all connectives to node i), and *s*_i_ is 1 if subject i is young and − 1 otherwise. A high *Q* indicates high observability of age-related differences, irrespective of the directions of the difference. Subsequently, the modularity index was calculated for each network and each denoising method. The values are then compared between denoising methods to understand their impact on the distinguishability of the two age groups. A higher modularity index indicates that the connectivity maps of young and old subjects are more distinguishable from each other. This does not imply a bigger distinction in neuronal activity between age groups, but serves to showcase the effect of each denoising method on detectable age-related differences.

#### Testing the generalizability of findings across sampling rates

To establish the generalizability of our results in highly sampled rs-fMRI data, we simulated the effect of conventional sampling (TR ~ 2 s) by downsampling the rs-fMRI data by a factor of 5, resulting in an effective TR of 1.9 s. We repeated the analyses on the resultant rs-fcMRI results (as power analysis on slowly-sampled fMRI data is not informative). While downsampling of data acquired at short TR likely does not represent the higher image SNR and tSNR of data acquired at longer TRs, this approach provides a “worst-case” representation of slowly sampled data.

#### Statistical testing

Both network-wise correlation and FCC (for both the original and downsampled data) were submitted to a mixed ANOVA with age group as the between-group factor and denoising method as the within-group factor. We executed follow-up *t*-tests for factors that displayed significance, employing paired t-tests between denoising methods and unpaired t-tests between age groups. All statistical analyses were performed using MATLAB (Version R2019b, Mathworks, Natick, United States).

## Results

**Figure 2:**
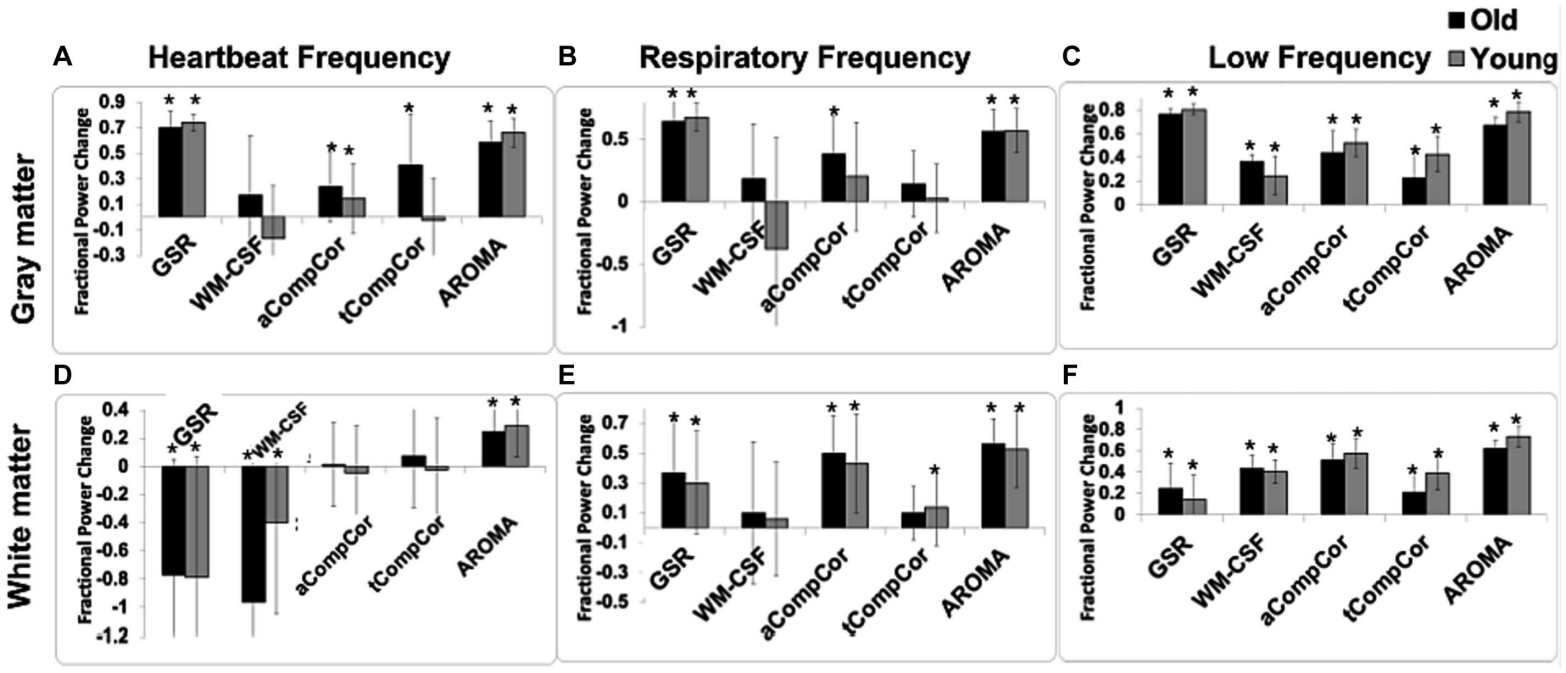
The effect of denoising strategies on the BOLD signal spectral power in young and older adults. Fractional power changes in cardiac **(A,D)**, respiratory **(B,E)**, and low-frequency bands **(C,F)** for the gray matter **(A–C)** and white matter **(D–F)** signals. Young and older adults are shown gray and black, respectively. Note that a positive power change represents a reduction in the spectral power due to denoising, and vice versa. Error bars represent the group-wise standard deviation. Asterisks indicate significant power differences from zero.

In Figure 2 are shown the fractional power changes in the cardiac-frequency, respiration-frequency and low-frequency bands for the whole GM (Figures 2A–C) and WM signals (Figures 2D–F). Results for young and older adults are shown in gray and black, respectively. In the GM, all denoising methods resulted in spectral-power reduction in all three frequency bands of the BOLD signal, removing as much as 60%–70% of the signal power in the respiratory and cardiac bands. GSR and AROMA removed the highest percentages of cardiac and respiratory BOLD-signal power, followed by aCompCor. However, GSR and AROMA also removed the largest percentages of the BOLD signal from the low-frequency band (up to 80%). In the WM, all methods appear less effective in removing cardiac power, with AROMA removing the most signal from (>70%). These differences are statistically significant, as shown in Table 1, which also shows there are significant age-method interactions in terms of power removed for all frequency bands except for the cardiac band.

**Table 1:** Results for multi-factorial ANOVA test using generalized linear mixed-effect models with fractional power change as the dependent variable, age, tissue type, and denoising method as factors.

Multi-factorial ANOVA results	Cardiac frequency		Respiratory frequency		Low frequency	
	F stat	*p*-value	F stat	*p*-value	F stat	*p*-value
By age	0.0076	0.93	0.022	0.88	0.387	0.53
By tissue type	86.59	**4.1e-18**	0.15	0.70	20.38	**3.4e-6**
By method	9.05	**7.0e-7**	12.40	**2.3e-9**	19.98	**1.2e-14**
Age-method interaction	1.15	0.33	2.40	**0.049**	5.25	**0.00040**

The ANOVA tests were conducted for fractional power changes in the respiratory, cardiac, and low-frequency bands separately. *p* values with those that are statistically significant shown in bold face.

Table 1 represents results for multi-factorial ANOVA tests with age, tissue type, and denoising method as factors. The results of the statistical tests are summarized below.

1. By frequency: young subjects showed significantly greater removal of low-frequency signals compared to old subjects. No significant difference was observed in the signal removed for cardiac and respiratory frequencies between young and old subjects.
2. By tissue type: the removal of signal in low and cardiac frequencies was significantly larger in the gray-matter compared to the white matter. There was no significant effect observed for the respiratory frequency band.
3. By denoising method: since the objective of this manuscript is to compare different denoising methods, a more comprehensive investigation of the denoising methods’ effect is conducted.

The ANOVA results also indicate that with the exception of power in the cardiac band, there are significant method x age interactions.

Taking Figure 2 and Tables 2A,B in conjunction, we observe that In young adults than older adults, for GM, GSR and AROMA removed significantly more signal power in the cardiac frequency band than all methods, and more signal power in the respiratory frequency band than all but WM-CSF regression. GSR and AROMA removed more low-frequency signal than all other denoising methods. For WM, AROMA removed the most power in the cardiac, respiratory and low-frequency bands, followed by aCompCor. GSR removed the least low-frequency power in the WM. GSR and WM-CSF regression introduced increases to cardiac power. For the older-adult data, WM-CSF regression performed worse, adding to rather than removing power from the cardiac and respiratory bands. Other trends are similar as in the young group. Moreover, across all noise bands, all methods appear to remove less noise power from the BOLD signals of young adults compared to those of older adults. Across both age groups, aCompCor and tCompCor performed similarly for removing signals in the cardiac frequency band, but aCompCor is more effective than tCompCor for reducing respiratory power.

**Table 2:** Pairwise comparisons of fractional power changes associated with different denoising methods.

(A)	Young												
	Cardiac					Respiratory				Low-frequency			
	*p* values	GSR	WM-CSF	aCompCor	tCompCor	GSR	WM-CSF	aCompCor	tCompCor	GSR	WM-CSF	aCompCor	tCompCor
**GM**	**WM-CSF**	**0.0004** ^ **<** ^				**0.0002** ^ **<** ^				**0.0002**^**<**^			
	**aCompCor**	**0.0004**^**<**^	**0.0262**^**>**^			**0.0003**^**<**^	**0.0002**^**>**^			**0.0002**^**<**^	**0.0009**^**>**^		
	**tCompCor**	**0.0004**^**<**^	0.5014	0.1089		**0.0002**^**<**^	0.0707	**0.0139**^**<**^		**0.0002**^**<**^	**0.0043**^**<**^	**0.0007**^**<**^	
	**AROMA**	**0.0437**^**<**^	**0.0004**^**>**^	**0.0004**^**>**^	**0.0004**^**>**^	**0.0096**^**<**^	**0.0002**^**>**^	**0.0002**^**>**^	**0.0002**^**>**^	0.3061	**0.0002**^**>**^	**0.0002**^**>**^	**0.0002**^**>**^
**WM**	**WM-CSF**	0.1337				**0.0084**^**<**^				**0.0084**^**<**^			
	**aCompCor**	**0.0019** ^ **>** ^	0.0787			0.0778	**0.0002**^**>**^			0.0778	**0.0002**^**<**^		
	**tCompCor**	**0.0027** ^ **>** ^	**0.0299** ^ **>** ^	0.7960		0.0854	0.3491	**0.0029**^**<**^		0.0854	0.3491	**0.0029**^**<**^	
	**AROMA**	**0.0004** ^ **>** ^	**0.0004** ^ **>** ^	**0.0019**^**>**^	**0.0072**^**>**^	**0.0038**^**>**^	**0.0003**^**>**^	**0.0429**^**>**^	**0.0003**^**>**^	**0.0038**^**>**^	**0.0003**^**>**^	**0.0429**^**>**^	**0.0003**^**>**^

(B)	Old												
	Cardiac					Respiratory				Low-frequency			
	*p* values	GS	WM-CSF	aCompCor	tCompCor	GS	WM-CSF	aCompCor	tCompCor	GS	WM-CSF	aCompCor	tCompCor
**GM**	**WM-CSF**	**0.0005**^**<**^				**0.0001**^**<**^				**0.0001**^**<**^			
	**aCompCor**	**0.0002**^**<**^	0.8926			**0.0023**^**<**^	0.0906			**0.0002**^**<**^	0.1353		
	**tCompCor**	0.0681	0.1909	0.0574		**0.0001**^**<**^	0.3258	**0.0017**		**0.0001**^**<**^	0.0676	**0.0031**^**<**^	
	**AROMA**	0.0942	**0.0002**^**>**^	**0.0002**^**>**^	0.1272	0.1937	**0.0006**^**>**^	**0.0001**^**>**^	**0.0001**^**>**^	**0.0017**^**<**^	**0.0001**^**>**^	**0.0001**^**>**^	**0.0001**
**WM**	**WM-CSF**	0.2734				**0.0085**^**<**^				**0.0166**^**>**^			
	**aCompCor**	**0.0024**^**>**^	**0.0007**^**>**^			0.2412	**0.0017**^**>**^			**0.0002**^**<**^	**0.0203**^**<**^		
	**tCompCor**	**0.0007**^**>**^	**0.0007**^**>**^	0.4973		**0.0166**^**>**^	0.7148	**0.0002**^**<**^		0.8077	**0.0017**^**>**^	**0.0001**^**<**^	
	**AROMA**	**0.0002**^**>**^	**0.0002**^**>**^	**0.0002**^**>**^	0.0574	0.0785	**0.0012**^**>**^	0.2676	**0.0001**^**>**^	**0.0001**^**>**^	**0.0002**^**>**^	**0.0040**^**>**^	**0.0001**^**>**^

Follow up comparisons for the multi-factorial ANOVA (Table 1) for **(A)** Data from young cohort; **(B)** data from older cohort. *p* values for GM and WM pairwise comparisons in different frequency bands, with those that are statistically significant shown in bold face. Underlining indicates where GSR and WM-CSF resulted in an increase rather than a decrease in fractional spectral power. “<” indicates where the denoising method on the row label removed a lower fractional power than the method on the column label, and “>” indicates the opposite.

**Figure 3:**
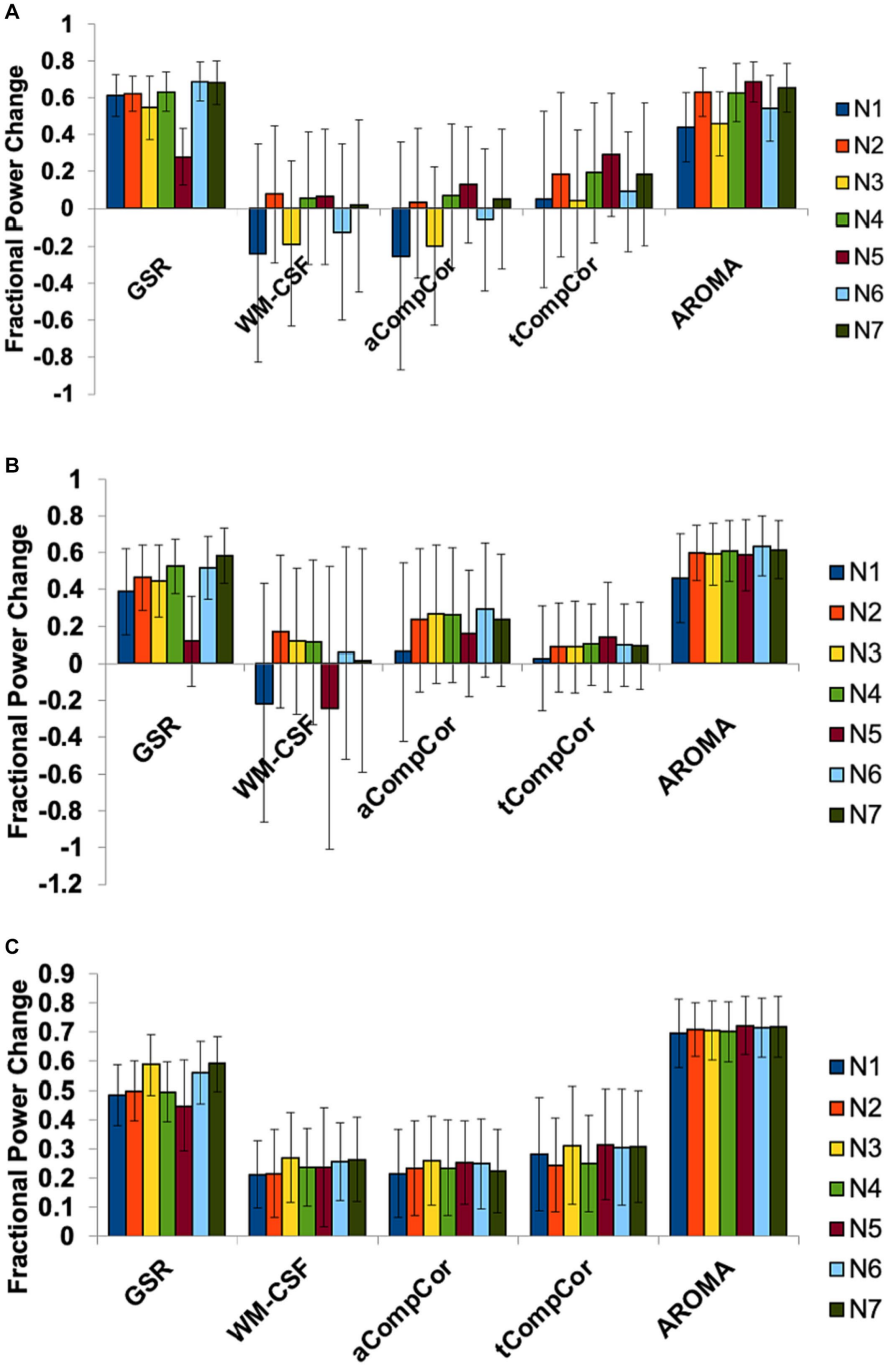
The effect of denoising strategies on the BOLD signal spectral power in different brain networks. Fractional power changes in **(A)** cardiac, **(B)** respiratory, and **(C)** low-frequency bands. N1: Visual, N2: Somatomotor, N3: Dorsal Attention, N4: Ventral Attention, N5: Limbic, N6: Frontoparietal, N7: DMN. Error bars represent the group-wise standard deviation.

We additionally performed spectral analysis for different networks to explore potential regional heterogeneity in the denoising performance. The results of this analysis are presented in Figure 3. The effects of denoising methods on spectral power in the three frequency bands are relatively uniform across all resting state networks.

As a surrogate of data quality, tSNR was found to be consistently higher with denoising than without, irrespective of the method (Supplementary Figure S2). Moreover, the tSNR associated with ICA-AROMA was significantly higher than those of all other methods examined. Also, we found the pre-denoising tSNR to be lower in the old subjects for Networks 2, 3, 4, and in the white matter region, as documented in Supplementary Tables S1, S2. In Network 5, the tSNR was lower in young subjects when compared to the old subjects. This indicates that, in general, older subjects exhibit lower tSNR. However, magnitudes of bulk head motion (through FD) and cardiac/respiratory contributions to the fMRI spectra (in the cardiac and respiratory bands) do not significantly differ between age groups (see Supplementary Table S3).

**Figure 4:**
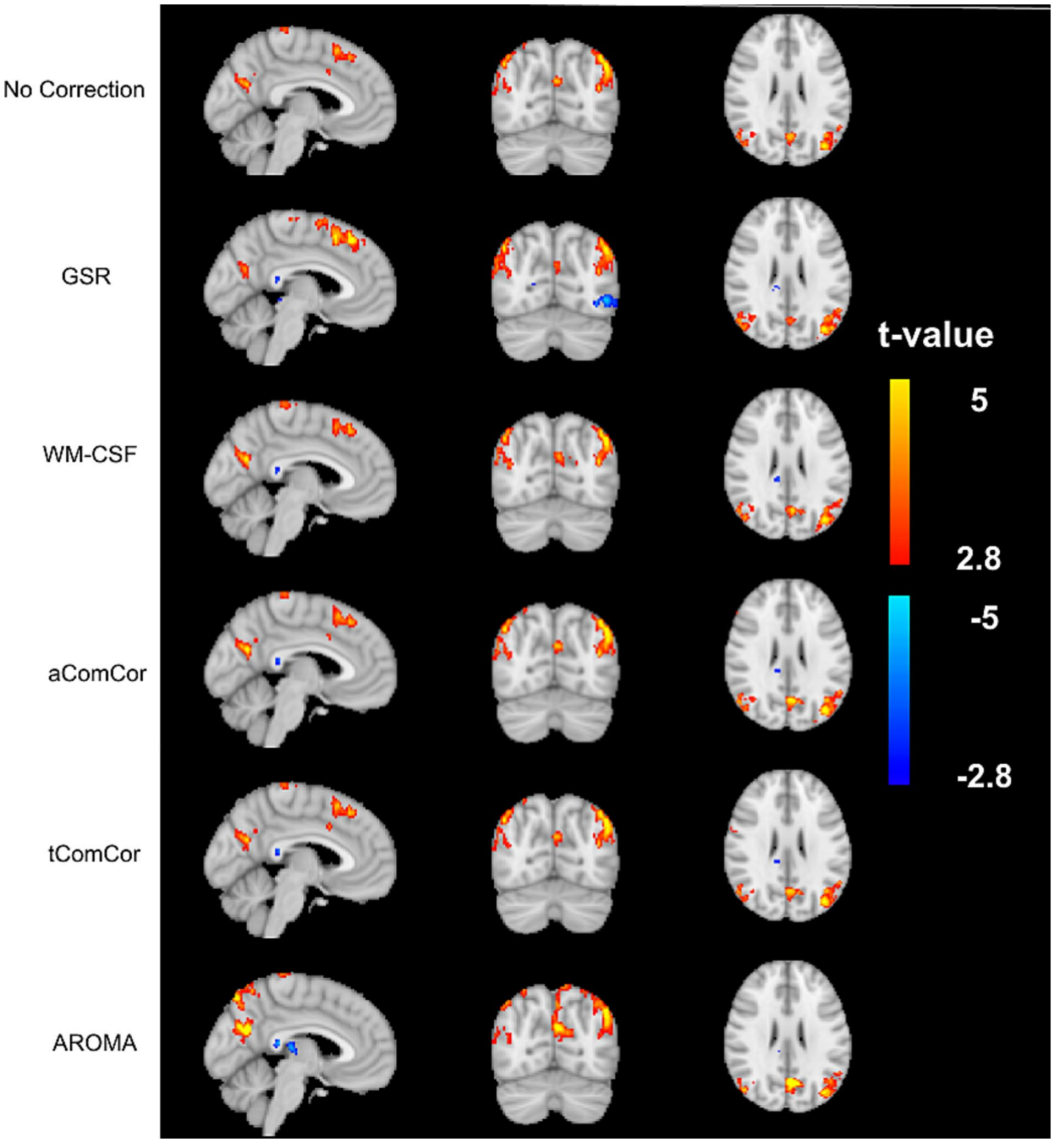
Sample age-related connectivity-difference maps for the default-mode network. Each row shows the difference *t*-map generated from each of the denoising methods. The color bar represents the range of *t*-values for the *t*-maps.

In Figure 4 we demonstrate the age-related fcMRI differences, shown here for the case of the default-mode network (DMN) using different denoising methods. Qualitatively, the maps generated from aCompCor, tCompCor and WM-CSF appear to be the most similar to each other. Quantitatively, values from the age-related fcMRI difference t-maps generated using different denoising methods are shown in different rows of Supplementary Figure S1, which shows that all denoising methods resulted in visually similar connectivity differences, with connectivity in older adults lower than in young adults (median *t*-value differences <0).

**Figure 5:**
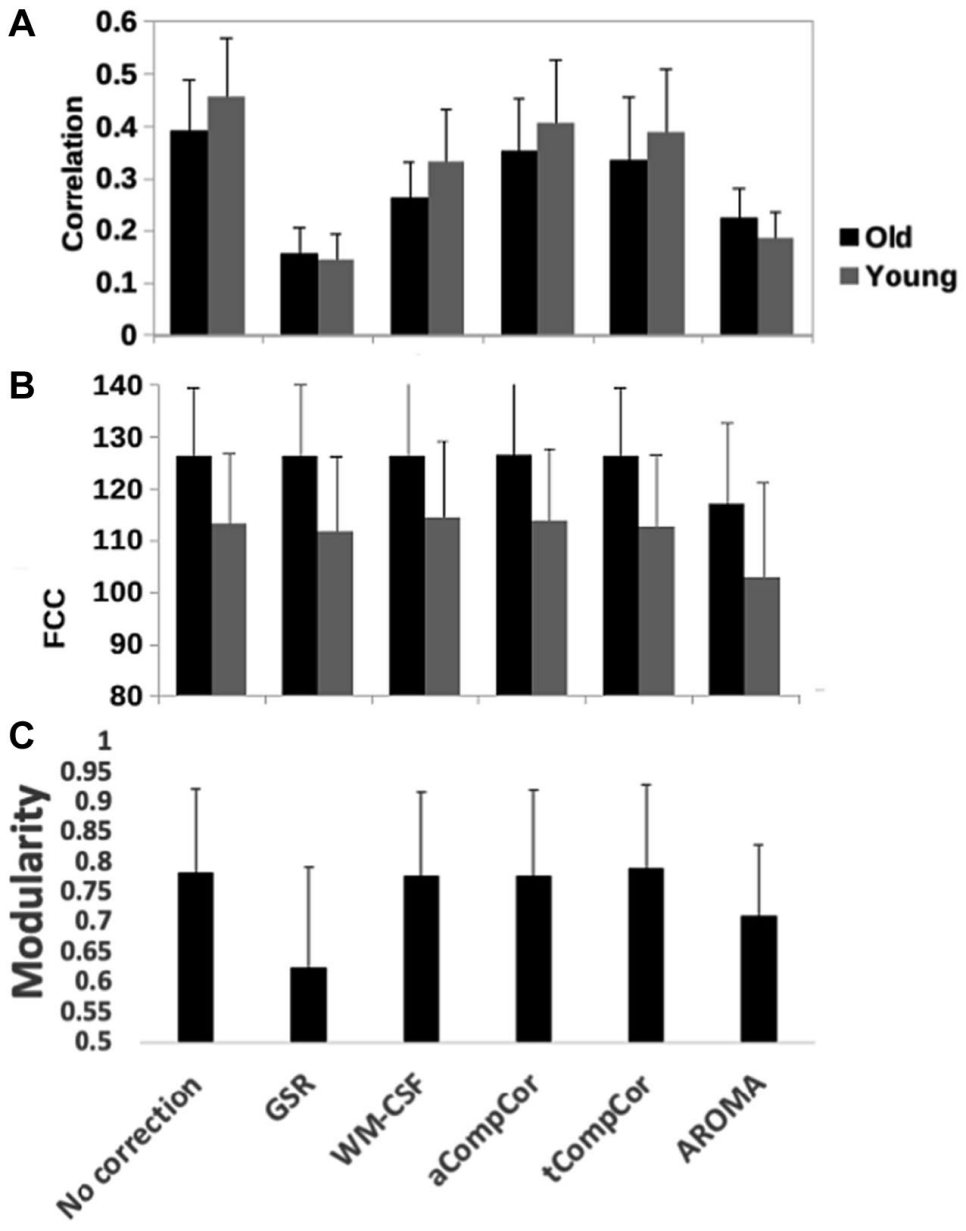
Quantitative comparison of rs-fMRI metrics across denoising methods, including **(A)** the network-mean Pearson’s correlation, **(B)** the functional-connectivity contrast (FCC) and **(C)** modularity index of the brain networks. GSR and AROMA methods considerably reduced modularity compared to other methods. Error bars represent standard deviation and significant differences, if any, are indicated by asterisks.

Figure 5 demonstrates the results of the age-related modularity and FCC analysis. There is no significant age-related difference in the correlation or FCC values. Statistical results of the pairwise comparisons in Table 3B show that the application of aCompCor resulted in significantly higher FCC than “no correction,” while WM-CSF and aCompCor are both associated with higher FCC than GSR. The application of AROMA denoising is associated with a substantial reduction in FCC. Conversely, the tCompCor, GSR, and WM-CSF denoising methods correspond to higher FCC. These differences were supported by the ANOVA results, which showed a significant effect of the denoising method on FCC (*p* = 2.5e-8). There were no significant effects of age (*p* = 0.096) and age-method interaction (*p* = 0.87) on FCC. Moreover, the ANOVA showed no significant age difference in mean Pearson’s correlation, but a significant effect of denoising method (*p =* 9.5e-44) and a significant age x method interaction (*p* = 0.0019). Furthermore, Figure 5C reveals that GSR and AROMA methods significantly reduce modularity, a metric that quantifies the observability of age-related differences. On the other hand, tCompCor exhibited the highest modularity values, followed by no-correction, aCompCor, and WM-CSF, as shown in Table 3C. The results corresponding to the downsampled data are shown in Supplementary Figure S3 and Supplementary Table S4. Taken together, the findings here appear to be consistent across the two and thus independent of fMRI sampling rate.

**Table 3:** Pairwise comparison of **(A)** Pearson’s correlation, **(B)** FCC associated and **(C)** modularity index with different denoising methods, corresponding to values in Figure 5.

(A)					
*p*-values	No correction	GSR	WM-CSF	aCompCor	tCompCor
GSR	5.1e-17^<^				
WM-CSF	3.8e-8^<^	2.0e-10^>^			
aCompCor	2.0e-8^<^	1.1e-13^>^	0.0002^>^		
tCompCor	5.0e-7^<^	9.2e-12^>^	0.0064^>^	0.0366^<^	
AROMA	4.8e-14^<^	1.8e-5^>^	8.9e-7^<^	6.7e-11^<^	1.7e-9^<^

(B)					
*p*-values	No correction	GSR	WM-CSF	aCompCor	tCompCor
GSR	0.05099				
WM-CSF	0.1935	0.0005^>^			
aCompCor	0.0080^>^	0.0003^>^	0.7591		
tCompCor	0.0483	0.1346	0.0358^<^	2.1e-7^<^	
AROMA	2.7e-7^<^	3.0e-7^<^	1.1e-9^<^	2.2e-8^<^	2.2e-7^<^

(C)					
*p*-values	No correction	GSR	WM-CSF	aCompCor	tCompCor
GSR	**0.0006**^**<**^				
WM-CSF	0.58	**0.0012**^**>**^			
aCompCor	0.14	**0.0004**^**>**^	0.96		
tCompCor	0.0467	**0.0003**^**>**^	0.26	**0.0073**^**<**^	
AROMA	**0.018**^**<**^	**0.023**^**>**^	**0.020**^**<**^	**0.021**^**<**^	**0.0092**^**<**^

The modularity index is compared across the seven networks between all pairs of denoising methods using paired *t*-test. The superscript “<” indicates where the value associated with the method in the row header is lower than that associated with the method in the column header, whereas the superscript “>” indicates the opposite. *p* values with those that are statistically significant shown in bold face.

## Discussion

In this work, we assessed the influence of a variety of popular data-driven denoising methods on resting-state BOLD signal-power and on rs-fcMRI maps in commonly observed resting-state functional networks. We specifically assess the impact of denoising choice on the resultant age-related fcMRI differences in these networks. Using a fMRI dataset acquired at high sampling rate, we were able to directly calculate the amount of spectral power altered by each method at each subject’s cardiac and respiratory frequencies. To summarize, in the GM, all methods reduced the BOLD spectral power in these physiological frequency ranges, albeit the degree of removed spectral power is significantly different among the methods, with GS and AROMA removing the most power. These methods, however, also removed the greatest amount of low-frequency power (0.1 Hz band). In contrast, CompCor (aCompCor and tCompCor) seems to result in an intermediate outcome in terms of both noise removal and retention of age-related differences. In the WM, all methods reduced the signal power in the respiratory frequency ranges. However, the performance of the methods in removing cardiac frequencies is more variable across subjects (see Figure 1). Comparisons on downsampled fMRI data delivered similar results.

### Physiological noise power spectrum

Cardiac pulsation and respiration are the two chief sources of physiological noise in the fMRI signal, especially problematic in rs-fMRI. Cardiac pulsations generate localized brain motion as well as creating inflow effects in and around blood vessels and CSF. On the other hand, thoracic movement during breathing results in magnetic field alteration and phase shift in the image. Moreover, respiration induces fluctuation in the level of arterial CO_2_, which has a vasodilating effect and can induce changes in the fMRI BOLD signal. The frequency of physiological signals in an adult human typically falls within the range of 0.2 to 0.3 Hz for respiratory and 0.8 to 1.2 Hz for cardiac signals. In a conventional fMRI scan with a TR of 2 s, these signals alias into the low-frequency range (0.01 to 0.1 Hz), which is known to contain information about fcMRI (Cordes et al., 2001). Physiological denoising in a conventional fMRI dataset may result in a decrease in signal power in the low-frequency range. However, it is impossible to determine whether the removed power is related to the respiratory and cardiac contributions or to neuronal connectivity. The low-frequency range of fMRI signals has signatures of several non-neuronal sources, including systemic low frequency oscillations (Tong et al., 2015, 2019), respiratory variations (Birn et al., 2006), and cardiac rate variations (Chang et al., 2009). Also, the fact that most of the denoising methods exhibit the highest impact on signal spectrum in the low-frequency band may be due to the nature of ROI-based noise regressor generation, whereby high-frequency regressor content may be averaged out through different phase shifts across the ROIs, leading to the retention of low-frequency regressor power.

### Choice of denoising methods

For context, the methods evaluated in this study are among the most commonly used in the resting-state fMRI literature. Specific to the study of the effect of aging on resting-state connectivity, all of the denoising methods have found broad application, including GSR (Betzel et al., 2014; Chan et al., 2014; Zhang et al., 2014; Geerligs et al., 2015; Siman-Tov et al., 2016; Stumme et al., 2020), WM-CSF (Koch et al., 2010; Betzel et al., 2014; Chan et al., 2014; Song et al., 2014; Zhang et al., 2014; Grady et al., 2016; Siman-Tov et al., 2016; Farras-Permanyer et al., 2019; Varangis et al., 2019; Mancho-Fora et al., 2020; Xie et al., 2020; Zhong and Chen, 2022), CompCor (Onoda and Yamaguchi, 2013; Hausman et al., 2020; Hamada et al., 2021; Patil et al., 2021; Podgórski et al., 2021), and ICA-AROMA (Stumme et al., 2020). Moreover, some recent studies of aging used no physiological denoising (Damoiseaux et al., 2008; Huang et al., 2015; Ao et al., 2022).

### Impact on BOLD signal spectrum

Owing to the high sampling rate of our fMRI data, we were able to dissect the differential effect of different denoising methods by examining the resultant fcMRI signal power spectrum. All methods resulted in spectral-power reduction in all three frequency bands of the GM signal, removing as high as 60–70% of the signal in the respiratory and cardiac bands. The amount of the removed power spectrum is vastly different among the methods, with GS and AROMA removing the most power followed by aCompCor. ICA-AROMA also removed more signal power in the physiological frequency bands compared to all other denoising methods. These findings are consistent with the finding of signal quality improvement by GSR and ICA-FIX by a previous study (Geerligs et al., 2017). Moreover, consistent with this finding, aCompCor was previously found to be less effective than ICA-FIX at reducing motion artifacts (Scheel et al., 2022).

In our findings, aCompCor and tCompCor removed significantly less power than GS and AROMA in all three frequency bands in both the GM and WM of both age groups, with only WM-CSF regression showing less power change (Tables 2A,B). This finding for aCompCor is in contrast to the findings of Agrawal et al. (2020) who found aCompCor to reduce low-frequency power while adding high-frequency noise power. Conversely, Muschelli et al., found aCompCor to remove more head-motion related signal contribution than alternatives such as WM-CSF regression (Muschelli et al., 2014). However, these latter findings were restricted to young adults, which suggests these discrepancies in findings may be related to the significance of age x denoising-method interactions reported in Table 1. Indeed, as reported in Supplementary Table S2, both mean and max FD were higher in the older adults. Nonetheless, no physiological frequency or head motion differences across age groups reached statistical significance.

Also, as mentioned earlier, Dipasquale et al. (2017) found ICA-AROMA to retained more low-frequency spectral power while reducing more high-frequency noise power than WM-CSF, which is in contrast to our findings. However, we wish to highlight that in order to isolate the effect of physiological-denoising, we did not regress out motion alongside the physiological noise regressors in this study, in contrast to previous studies (Dipasquale et al., 2017; Geerligs et al., 2017; Kassinopoulos and Mitsis, 2022). Thus, we cannot directly compare our results to those of Dipasquale et al., which included WM-CSF alongside motion regression. The interaction between motion and noise is complex (Jones et al., 2008), and the order of each type of correction as well as the number of motion regressors can sway the results (Ciric et al., 2017; Parkes et al., 2018), and thus these questions are beyond the scope of this work. Moreover, additional differences in findings could be attributed to the major differences in the TR of the BOLD acquisition. In the Dipasquale study, TR was 2.75 s, while in our study, it was 0.38 s. This difference in BOLD signal sampling rate could affect the performance of regression-based approaches such as WM-CSF, which do not discriminate between different temporal and spatial constituents in the WM and CSF ROIs. The evaluation of the effects of sampling rate and head motion on different denoising methods is a promising future direction.

Compared to the GM, the results in the WM are more variable across subjects, and WM denoising is much less examined in the literature. Notably, among all the methods, only AROMA appeared to reduce cardiac power (Figure 1D). All methods reduced power in the respiratory and low-frequency frequency bands (Figures 1E,F), with aCompCor and AROMA leading the other methods in this regard. Nevertheless, the power-reduction variability in the WM is much larger than that in GM, especially for GSR, WM-CSF and aCompCor. GSR is also very effective at suppressing the contribution of physiological frequencies in the GM, but not very effective in the WM. Unsurprisingly, both GSR and WM-CSF regression introduced increases to cardiac power, and are thus inappropriate for WM fMRI denoising. This observation could be attributed to the manner in which the noise regressors are derived for these methods, in which the WM signal is indiscriminately designated as noise. Interestingly, WM-CSF did not significantly reduce WM BOLD signal power in the cardiac and respiratory power, although this method can be viewed as involving regressing the WM signal out of itself. Although aCompCor also derives regressors from WM, its performance is superior to that of WM-CSF, as it only designated the leading principal components of the WM signal (and CSF) signal as noise regressors (Figure 1E).

### Interaction between head motion, fast sampling and the BOLD signal spectrum

It has been established that head-motion effects interact with physiological denoising (Jones et al., 2008) and age-related fcMRI differences (Mowinckel et al., 2012; Geerligs et al., 2017). Lesser known, but critical, is the additional influence of fMRI sampling rate, especially with the advent of fast fMRI (Chen et al., 2019; Power et al., 2019). We found a sub-significant age-group difference in head motion (FD), with older adults associated with higher FD (Supplementary Table S2).

Given our high sampling rate, we will elaborate on the unique effect of fast sampling on the treatment of head motion and the measurement of denoising outcome. In the acquisition used in this study, we used an SMS acceleration factor of 3 and 21 slices at TR = 0.38 ms, translating into a maximum samplable frequency of 1.3 Hz. This frequency is sufficient to capture the fundamental cardiac frequency, preventing its aliasing into lower-frequency bands. The SMS acquisition scheme also results in the shuffling of slice acquisition ordering across 7 slice groups. If residual head motion chiefly manifests as through-plane movement between neighboring voxels, then all slices in each slice group would shift simultaneously, expanding the influence of motion across the brain volume. Additionally, Power et al. (2019) reported on lower-frequency motion being subdivided by fast sampling (TR of 720 ms) and thus appearing as lower-amplitude higher-frequency FD that overlaps in frequency with respiratory noise. This effect of fast sampling is further complicated by slice groups being ordered in an interleaved fashion. That is, the slice group ordering was [1, 8, 15], [3, 10, 17], [5, 12, 19], … [2, 9, 16], [4, 11, 18], and so on, such that physiological and motion effects that should be similar across neighboring slices are sub-sampled at up to TR/2 apart. Each slice group would thus sub-sample the head-motion time course with a different phase shift, resulting in potentially elevated within-ROI signal heterogeneity for methods such as GSR and WM-CSF regression.

Relating this to different sampling rates, note that the use of a TR of 720 ms in Power et al.’s work sets the maximum samplable BOLD signal frequency at 0.7 Hz. At this sampling rate, the cardiac frequency (typically ~1 Hz) would alias into the 0.3 Hz frequency band, which coincides with the typical respiratory frequency. Thus, manifestation of fast-sampled motion, and consequently the effect of different denoising methods on the fast-sampling related motion-subsampling effects, which has previously been reported (Burgess et al., 2016; Ciric et al., 2017; Dipasquale et al., 2017), also depends on the actual sampling frequency and SMS factor.

### Impact on fcMRI strength

The low-frequency band’s interpretation remains complex, however. On the one hand, it is the established spectral location for the majority of the neurovascular contribution to brain functional connectivity (Cordes et al., 2001), and retention of fcMRI strength has been used as a metric for evaluating retention of meaningful BOLD signal (Dipasquale et al., 2017). Nonetheless, variability in the respiratory and cardiac pattern can also induce BOLD signal variations in the low-frequency frequency band (Wise et al., 2004; Birn et al., 2006; Chang et al., 2013). Recent work, however, demonstrated that respiratory-volume variability may have neuronal associations (Yuan et al., 2013; Shams et al., 2021), rendering its interpretation uncertain.

Our results showed that GSR and AROMA removed the most low-frequency BOLD signal power. Although low-frequency power did not significantly modulate GSR-based functional connectivity (Pearson’s correlation) or FCC as a measure of network segregation (Kassinopoulos and Mitsis, 2022), GSR and AROMA resulted in two of the lowest FCC values in comparison to the other methods (see Table 3B). Our downsampled data (Supplementary Figure S3 and Supplementary Table S4) followed very similar trends as the original data, suggesting that our findings are generalizable to different sampling rates, and that the effects of band-limited noise, once shifted to different frequency bands due to aliasing, may retain the same impact on functional connectivity metrics.

Moreover, AROMA-based Pearson’s correlation was significantly mediated by both cardiac and low-frequency power (Supplementary Table S5A). In contrast, WM-CSF and aCompCor were associated with the highest FCC values as demonstrated in (Figure 5B; Table 3B). Still, based on the power spectral analysis alone, it is impossible to understand whether the removed low-frequency spectrum can alter brain connectivity as measured by rs-fMRI. Furthermore, it is possible that a higher fcMRI measurement does not result from higher low-frequency power or lower noise power (Agrawal et al., 2020). Lastly, the reduced observable age-related differences in fcMRI measures associated with GSR and AROMA was not significantly mediated by spectral-power changes in any of the three frequency bands (Supplementary Table S5C), despite strong correlations between low-frequency power change and modularity in both GSR and AROMA (0.46 and − 0.74, respectively). Interestingly, in aCompCor alone, was spectral power change significantly associated with modularity index (positively for respiratory power change, and negatively for both cardiac and low-frequency power). These correlations are reflected also in the findings for tCompCor and AROMA, suggesting that these methods may share the property whereby higher age-related differences in connectivity may be linked to lower reductions in low-frequency and cardiac power and greater reduction in respiratory power. This is an interesting scenario for future investigations.

### Impact on age-related fcMRI differences

We also evaluated the denoising methods based on their effects on the observable age-related rs-fcMRI differences. Although aCompCor and tCompCor did not result in the greatest retention of low-frequency BOLD signal power (Figure 2; Table 2), they generated maps with the highest modularity indices (a measure of sensitivity to age-related differences), as demonstrated in Figure 5A and Table 3A. Moreover, we observe a significant age x denoising-method interaction when examining the fractional spectral power changes in the low-frequency and respiratory bands (Table 1).

In aging studies of fcMRI, rs-fcMRI reduction in the DMN is the most commonly reported finding [for a review please refer to Jockwitz and Caspers (2021)], although the extent and location of the connectivity reduction differ among the studies, consistent with our finding (Figure 2). For instance, Stumme et al. (2020) used AROMA denoising and reported reduced connectivity in the visual network and no changes in the DMN and somatomotor networks, whereas other studies that used tCompCor or WM-CSF denoising have reported no change (Zhang et al., 2014) or reduced (Onoda and Yamaguchi, 2013; Chan et al., 2014; Zhang et al., 2014) connectivity in the visual network and reduced connectivity in the DMN (Onoda and Yamaguchi, 2013; Betzel et al., 2014; Chan et al., 2014; Zhang et al., 2014; Grady et al., 2016), as well as no connectivity change in the somatomotor network (Onoda and Yamaguchi, 2013; Betzel et al., 2014; Chan et al., 2014). Here, we demonstrate that one possible source of the inconsistencies across studies is the choice of method for physiological denoising. Despite the fact that the spectral power removed by different denoising methods were at times not significantly different, these differences resulted in observable differences in the resultant observable age-related fcMRI differences. Specifically, as shown in Figure 5A and Table 3A, the GSR method also visibly reduced the observable age difference in fcMRI, while CompCor and WM-CSF minimally impacted the observable age difference in connectivity. Nonetheless, the ability to detect age differences does not equate that of detecting neuronally-driven age differences.

In the systematic comparison of WM-CSF and aCompCor reported by Geerligs et al. (2017), WM-CSF regression was associated with higher connectivity strength and reliability as well as reliability of age effects than aCompCor. Moreover, Geerligs et al. showed that the WM-CSF regression resulted in a negative age-fcMRI association, while aCompCor resulted in a positive age-fcMRI association. We did not test for reliability of our fcMRI metrics, preventing a direct comparison of results in that regard. However, we did not observe a significant difference in modularity index between WM-CSF and aCompCor.

### Limitations

This study presents several limitations. Firstly, we focused only on the denoising methods implemented in fmriprep. More advanced methods have been developed recently (Agrawal et al., 2020; Shin et al., 2022; Bancelin et al., 2023) wechose to compare only the methods implemented in fmriprep, as they may be more commonly used. The same methodology can be used to compare other physiological denoising techniques. Moreover, we only evaluated the methods in their ability to remove physiological signals. The methods can perform differently in removing other types of noise. For example, ICA-AROMA has been shown to be more successful in removing head motion compared to aCompCor (Pruim et al., 2015a). The removed head motion may affect high-frequency as well as low-frequency bands, depending on the head motion type. Low-frequency head motion has been associated with rhythmic motion related to breathing (Power et al., 2019), and may be addressed by physiological regression, while random abrupt motion can be a more broadband disruption that is not captured in physiological noise time courses. In fact, these high-frequency motion signatures have also been associated with useful between-group effects (Yuan et al., 2016).

Secondly, in the absence of a ground-truth, we simply showcase a comparison of different denoising methods for revealing the age-related fcMRI differences. Indeed, head motion (both bulk and localized) differs between age groups, with older adults known to exhibit greater degrees of movement (Van Dijk et al., 2012; Pardoe et al., 2016; Savalia et al., 2017; Madan, 2018; Saccà et al., 2021), although in this study head motion is not significantly different between our young and old subjects (see Supplementary Table S2). Furthermore, we did not include head motion as regressor in our pipeline, fully recognizing the likely complex interactions between motion regression and denoising. Thus, a bigger separation between ages does not imply greater ability to detect functional differences, but may rather serve as a marker of age-related difference, which has applications in its own right. Moreover, we did not formally test BOLD fractional power change in any of the three frequency bands as a mediator of the fcMRI differences associated with the different denoising methods.

Thirdly, we did not explicitly remove head motion as we did not record head motion independently of the image acquisition. The effect of bulk and regional head motion can be profound to our ability to discern neuronally specific connectivity patterns, and will be the focus of our future work.

Lastly, hybrid approaches that combine the test methods are becoming increasingly recognized (Li et al., 2019; Van Schuerbeek et al., 2022; Weiler et al., 2022). While a thorough investigation of these potentially powerful combinations is beyond the scope of this work, we trust that in order to effectively combine different methods, it is even more important to understand the effect of each denoising method in isolation. Alternatively, a multiverse approach (Dafflon et al., 2020; Chen et al., 2023) can be used to systematically explore a diverse range of denoising methods and explore the robustness and reproducibility of findings corresponding to different methodological choices.

## Conclusion

In this study, we compared the outcomes of several data-driven noise removal methods in altering power spectra in the physiological noise and low-frequency bands, as well as their impact on age-related differences in functional connectivity. GSR and AROMA excel in removing signals in frequency bands corresponding to cardiac and respiratory frequencies, but were also associated with lower age-related connectivity differences. In comparison, aCompCor and tCompCor were associated with greater observable age-related connectivity differences. It is important to note that not all age-related differences are driven by neuronal differences, and that the denoising methods tested in this work performed differently for different age groups. The roles of head motion and sampling rate are important considerations when comparing denoising methods.

## Data availability statement

The raw data supporting the conclusions of this article will be made available by the authors, without undue reservation.

## Ethics statement

The studies involving humans were approved by Baycrest Research Ethics Board. The studies were conducted in accordance with the local legislation and institutional requirements. The participants provided their written informed consent to participate in this study.

## Author contributions

AG: conceptualization, data acquisition, data analysis, and manuscript preparation. JC: data acquisition, supervision of data analysis, and manuscript preparation. All authors contributed to the article and approved the submitted version.
