# Supplementary material for: Comparing data-driven physiological denoising approaches for resting-state fMRI: implications for the study of aging
Source: Front Neurosci. 2024 Feb 6;18:1223230. doi: 10.3389/fnins.2024.1223230 (PMC10876882; doi:10.3389/fnins.2024.1223230)
Supplement: Supplementary file 1 [file Data_Sheet_1.docx]

### Functional connectivity across networks and denoising methods


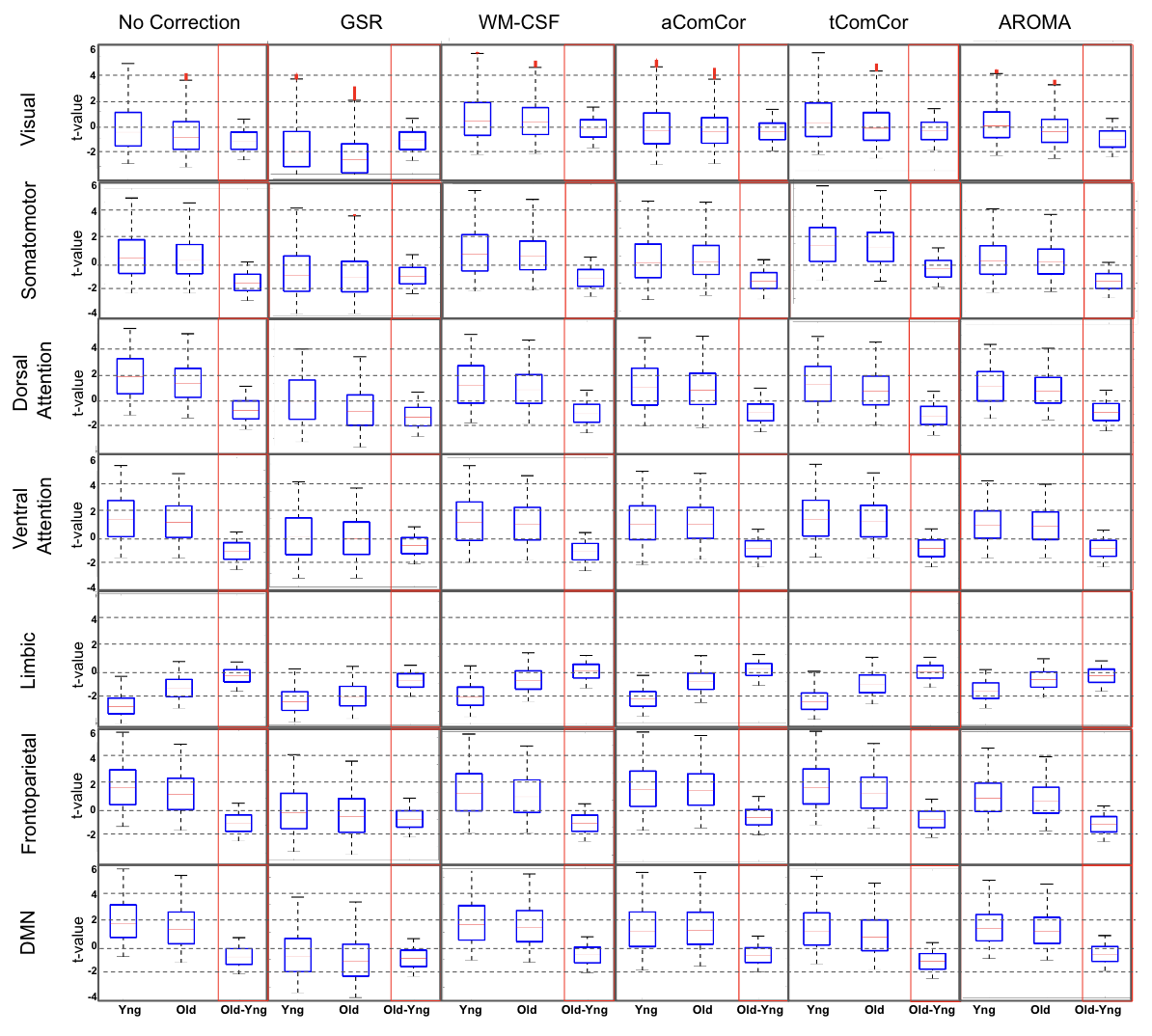


**Figure S1. Comparison of fcMRI measurements corresponding to different denoising methods and functional networks.** The network-specific average t values are represented as box plots generated from the 7 network templates defined by the Yeo fcMRI atlas, labeled along the vertical axis. The fcMRI t-value distributions are plotted for the young (Yng), older (Old) and age difference (Old-Yng), whereby the error bars represent the full extent of the distribution. All denoising methods resulted in visually similar connectivity differences, with connectivity in older adults lower than in young adults (median t-value differences < 0). DMN = default-mode network.

### Temporal SNR

The temporal signal to noise ratio (tSNR) was calculated as the average intensity of each voxel divided by its standard deviation. The tSNR values were averaged within each network, as well as in the white matter. Subsequently, the tSNR values were compared between young and old subjects, as well as across denoising methods. The results of the comparison showed that tSNR was lower in the old subjects. Moreover, AROMA considerably increased tSNR in both old and young subjects.


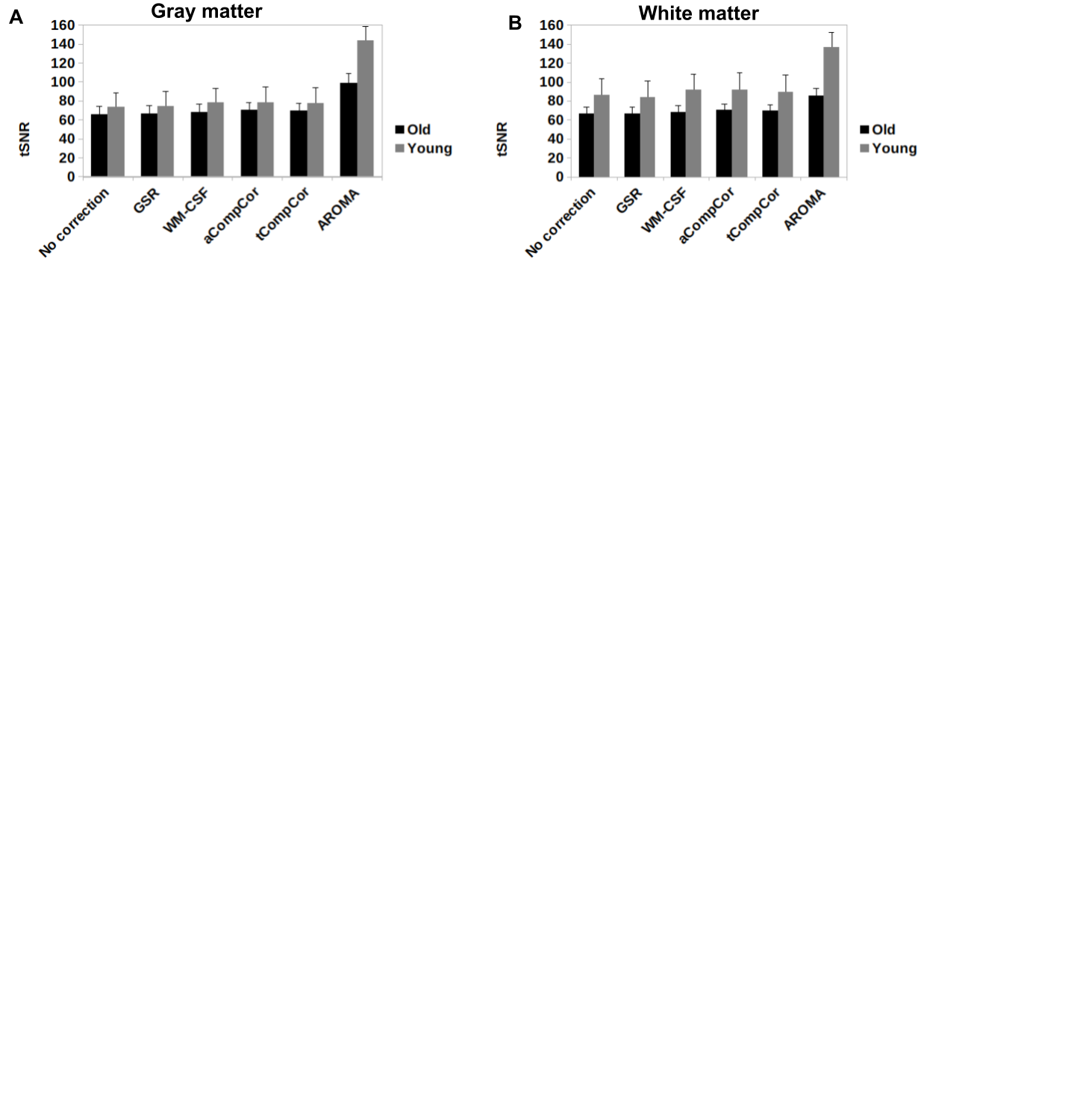


**Figure S2. temporal SNR compared between young and old subjects across different denoising methods for both gray matter and white matter**.

**Table S1. Comparison of the tSNR between young and old subjects and denoising methods.** P-values associated with significant differences after FDR correction are shown in bold. The superscript ‘<’ indicates where the modularity index associated with the method in the column header is lower than that associated with the method in the row header, whereas the superscript ‘>’ indicates the opposite.

ANOVA results for the gray matter:

Age effect: F = 10.752, p = 0.0026

Method effect: F = 602.15, p = 6.98e-25

|  | Old | Young | p-value |
| --- | --- | --- | --- |
| tSNR | 73.04 | 87.62 | **0.0016** |

| P-values | GSR | WM-CSF | aCompCor | tCompCor | AROMA |
| --- | --- | --- | --- | --- | --- |
| No Correction | **6.4e-6^>^** | **1.1e-6^>^** | 0.0809 | **0.0007^>^** | **3.4e-14^>^** |
| GSR |  | **7.6e-10^>^** | **5.1e-12^>^** | 0.6126 | **6.1e-14^>^** |
| WM-CSF |  |  | **3.5e-15^>^** | **1.2e-11^>^** | **5.9e-15^>^** |
| aCompCor |  |  |  | **9.7e-1^<^** | **6.4e-15^>^** |
| tCompCor |  |  |  |  | **3.4e-15^>^** |

ANOVA Results for the white matter:

Age effect: F = 29.987, p = 6.1e-6

Method effect: F = 373.03, p = 2.5e-24

|  | Old | Young | p-value |
| --- | --- | --- | --- |
| tSNR | 71.13 | 96.73 | **3.5e-6** |

| P-values | GSR | WM-CSF | aCompCor | tCompCor | AROMA |
| --- | --- | --- | --- | --- | --- |
| No Correction | 0.0018 | **2.4e-7^>^** | 0.2456 | **4.4e-8^>^** | **8.5e-12^>^** |
| GSR |  | **1.3e-7^>^** | **1.5e-10^>^** | 0.2483 | **1.8e-11^>^** |
| WM-CSF |  |  | **1.8e-13^>^** | **3.5e-10^<^** | **8.9e-13^>^** |
| aCompCor |  |  |  | **6.8e-15^<^** | **2.0e-12^>^** |
| tCompCor |  |  |  |  | **1.4e-12^>^** |

### Age-related differences in physiological noise and head motion

**Table S2. comparison of the cardiac and respiratory frequencies as well as framewise displacement of subjects from the young and old groups using unpaired t-tests.**

|  | **Old** | **Young** | **p-value** |
| --- | --- | --- | --- |
| **Cardiac Frequency** | 1.10 ±0.15 | 1.11 ±0.19 | 0.89 |
| **Respiratory Frequency** | 0.24±0.33 | 0.24±0.06 | 0.94 |
| **Mean FD** | 0.0052±0.0078 | 0.0015±0.00060 | 0.051 |
| **Max FD** | 0.044±0.063 | 0.025±0.016 | 0.23 |

**Table S3. Correlation between head motion (FD) and signal power in different frequency bands in the gray matter and white matter.** The table shows that there is no significant association between bulk head motion and physiological signals.

|  |  | **Correlation** | **p-value** |
| --- | --- | --- | --- |
| **Cardiac** | GM | -0.090 | 0.6415 |
|  | WM | -0.032 | 0.8687 |
| **Respiratory** | GM | -0.077 | 0.6763 |
|  | WM | -0.006 | 0.975 |
| **Low Frequency** | GM | -0.063 | 0.7336 |
|  | WM | 0.046 | 0.8011 |

### Cross-validation with downsampled data


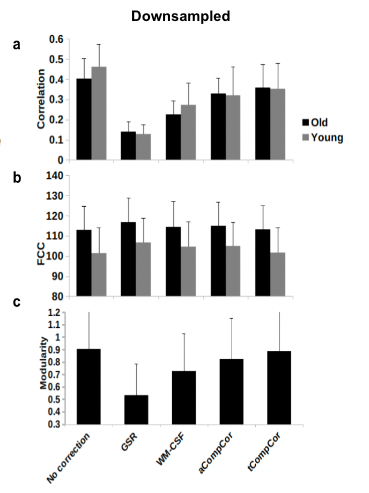


**Figure S3. Quantitative comparison of rs-fMRI metrics across denoising methods (down-sampled),** including (a) the network-mean Pearson’s correlation, (b) the functional-connectivity contrast (FCC) and (c) modularity index of the brain networks**.** GSR and AROMA methods considerably reduced modularity compared to other methods. Error bars represent standard deviation and significant differences, if any, are indicated by asterisks.

**Table S4. Pairwise comparison of (a) Pearson’s correlation, (b) FCC associated and (c) modularity index with different denoising methods, corresponding to values in** **Fig. S3**. The modularity index is compared across the seven networks between all pairs of denoising methods using paired t-test. The superscript ‘<’ indicates where the value associated with the method in the row header is lower than that associated with the method in the column header, whereas the superscript ‘>’ indicates the opposite.

(a)

| p-values | No correction | GSR | WM-CSF | aCompCor |
| --- | --- | --- | --- | --- |
| GSR | 3.8e-17^<^ |  |  |  |
| WM-CSF | 1.7e-12^<^ | 3.5e-9^>^ |  |  |
| aCompCor | 8.0e-9^<^ | 5.0e-11^>^ | 0.0012^>^ |  |
| tCompCor | 3.2e-5^<^ | 4.1e-12^>^ | 4.5e-5^>^ | 0.0225^<^ |

(b)

| p-values | No correction | GSR | WM-CSF | aCompCor |
| --- | --- | --- | --- | --- |
| GSR | 2.6e-12^>^ |  |  |  |
| WM-CSF | 1.7e-5^>^ | 3.5e-7^<^ |  |  |
| aCompCor | 2.5e-6^>^ | 0.0002^<^ | 0.3348 |  |
| tCompCor | 0.3580 | 6.8e-12^<^ | 4.6e-6^<^ | 2.4e-9^<^ |

(c)

| p-values | No correction | GSR | WM-CSF | aCompCor |
| --- | --- | --- | --- | --- |
| GSR | **0.0028^<^** |  |  |  |
| WM-CSF | 0.0024**^<^** | **0.0047^>^** |  |  |
| aCompCor | 0.0015**^<^** | **0.0004^>^** | 0.0133**^>^** |  |
| tCompCor | 0.0689 | **0.0025^>^** | 0.0024**^>^** | **0.0015^<^** |

### Mediation analysis

We test for mediation effects between spectral power and rs-fMRI metrics. Spectral power changes in the cardiac, respiratory and low-frequency bands are entered simultaneously as independent variables in a partial-correlation analysis to explain observed differences in all rs-fMRI metrics resulting from all denoising methods. The changes in the rs-fMRI metrics are calculated with respect to the no-correction method, similar to the approach described in Eqn 1. The partial correlation analysis shows the association between the changes in the power of each frequency band and the changes observed in the rs-fMRI metrics, while accounting for the effect of the other two frequency bands. We averaged the values for Pearson’s correlation and FCC across the seven networks and subsequently calculated the correlation across subjects. However, the calculation of modularity generates one value from all subjects. Therefore, it is not feasible to calculate a correlation between modularity and power spectrum across subjects. To circumvent this limitation, we evaluated the association between modularity and spectral power changes across the seven networks.

Based on partial correlation analysis, spectral power changes due to denoising widely reduced Pearson’s correlation values, with those associated with GSR and AROMA being consistently the lowest (**Table S5a**). For WM-CSF and AROMA, cardiac and low-frequency power both significantly modulated correlation coefficients, whereas for aCompCor, it was respiratory power alone, and for tCompCor, it’s low-frequency power alone. Additionally, there is no significant contribution to FCC variations by spectral power variations for most methods (**Table S5b)**. The only exception is for tCompCor, in which cardiac and low-frequency fractional power changes contribute significantly to FCC. Likewise, pertaining to the influence of power-spectral change on detectable age-related differences (modularity index) (**Table S5c)**, most methods did not exhibit a significant correlation. However, aCompCor and WM-CSF are two exceptions. In the former case, the modularity index was significantly correlated with all three spectral powers, while for the latter, modularity was significantly correlated with respiratory power. These findings in part support spectral power as mediating observable age-related differences in FC, for the case of aCompCor and WM-CSF.

**Table S5. Partial-correlation analysis relating spectral power to rs-fMRI metrics. (a) Pearson’s correlation, (b) FCC associated and (c) modularity index with different denoising methods.**

(a)

| Pearson’s  Correlation | Cardiac | | Respiratory | | Low Frequency | |
| --- | --- | --- | --- | --- | --- | --- |
|  | r | p | r | p | r | p |
| GSR | 0.23 | 0.24 | -0.27 | 0.17 | 0.31 | 0.11 |
| WM-CSF | **0.42** | **0.030** | -0.18 | 0.38 | **0.60** | **0.00084** |
| aCompCor | 0.27 | 0.18 | **0.47** | **0.015** | 0.38 | 0.052 |
| tCompCor | 0.34 | 0.084 | -0.034 | 0.87 | **0.73** | **1.27e-05** |
| AROMA | **0.64** | **0.00033** | -0.33 | 0.10 | **0.81** | **3.28e-07** |

(b)

| FCC | Cardiac | | Respiratory | | Low Frequency | |
| --- | --- | --- | --- | --- | --- | --- |
|  | r | p | r | p | r | p |
| GSR | -0.32 | 0.10 | 0.12 | 0.5 | -0.24 | 0.22 |
| WM-CSF | -0.27 | 0.17 | -0.12 | 0.55 | 0.26 | 0.20 |
| aCompCor | -0.38 | 0.047 | -0.072 | 0.72 | -0.20 | 0.31 |
| tCompCor | **-0.57** | **0.0010** | -0.053 | 0.79 | **-0.41** | **0.032** |
| AROMA | -0.086 | 0.67 | 0.061 | 0.76 | 0.38 | 0.052 |

(c)

| Modularity | Cardiac | | Respiratory | | Low Frequency | |
| --- | --- | --- | --- | --- | --- | --- |
|  | r | p | r | p | r | p |
| GSR | 0.77 | 0.13 | -0.70 | 0.18 | 0.46 | 0.43 |
| WM-CSF | 0.52 | 0.37 | **-0.90** | **0.037** | 0.82 | 0.090 |
| aCompCor | **-0.99** | **0.00053** | **0.98** | **0.0043** | **-0.99** | **0.0015** |
| tCompCor | 0.58 | 0.30 | -0.62 | 0.26 | -0.31 | 0.61 |
| AROMA | -0.26 | 0.67 | 0.63 | 0.25 | -0.74 | 0.15 |

## 
